# The prognostic significance of nuclear expression of PHF2 and C/EBPα in clear cell renal cell carcinoma with consideration of adipogenic metabolic evolution

**DOI:** 10.18632/oncotarget.19949

**Published:** 2017-08-04

**Authors:** Jeong Hwan Park, Minsun Jung, Kyung Chul Moon

**Affiliations:** ^1^ Department of Pathology, Seoul National University College of Medicine, Seoul, Republic of Korea; ^2^ Department of Pathology, SMG-SNU Boramae Medical Center, Seoul, Republic of Korea; ^3^ Kidney Research Institute, Medical Research Center, Seoul National University College of Medicine, Seoul, Republic of Korea

**Keywords:** PHF2, C/EBPα, clear cell renal cell carcinoma, adipogenesis, progression

## Abstract

Clear cell renal cell carcinoma (ccRCC) is the most common subtype of renal cell carcinoma (RCC), and it has an unfavourable prognosis compared to other RCCs. Plant homeodomain finger 2 (PHF2) and CCATT/enhancer binding protein α (C/EBPα) play a role in the epigenetic regulation of adipogenesis, and their tumour suppressive functions have been elucidated. This study aimed to assess the nuclear expression of PHF2 and C/EBPα in ccRCC and to evaluate their role in pathogenesis and prognosis. The nuclear expression of PHF2 and C/EBPα was evaluated in 344 cases of ccRCC by immunohistochemistry, and adipogenesis was assessed based on cytoplasmic features. Low expression was significantly associated with a larger tumour size, higher WHO/ISUP grade, high pT, pM, and advanced pTNM stage. Additionally, the expression level was correlated with the cytoplasmic features of ccRCC. The low expression group had significantly shorter cancer-specific and progression-free survival times. Furthermore, multivariate analysis showed that the combination of PHF2 and C/EBPα expression as an independent prognostic factor for cancer-specific and progression-free survival. In conclusion, our results suggest that nuclear expression of PHF2 and C/EBPα may serve as a prognostic marker and that the oncogenic metabolic shift has progressed in ccRCC patients.

## INTRODUCTION

Renal cell carcinoma (RCC) is the most common cancer of the kidney and is amongst the most lethal urinary malignancies [1]. Patients with RCC presented with metastatic disease at rates of 30% and higher, and approximately 40% had local recurrence or distant metastases develop during follow-up [2, 3]. Among various subtypes of RCC, clear cell RCC (ccRCC) is the most common histologic subtype, accounting for 65 to 70% of all renal malignancies, and it has a less favourable prognosis than other RCC subtypes [4]. ccRCC metastasis mainly occurs *via* haematogenous spread, and the lungs, bone, and liver (in order) are common metastatic sites [5]. The outcome of metastatic RCC is dismal, and the 5-year survival of metastatic RCC is estimated to be less than 10% [6].

The molecular characteristics of ccRCC have been elucidated with the development of molecular studies including the next-generation sequencing (NGS) technique [4, 7, 8]. In considering the genetic alteration levels, mutations in the *Von Hippel-Lindau* (*VHL*) gene, which acts as tumour suppressor gene by stabilizing hypoxia inducible factors (HIF-1α and HIF-2α) and the *polybromo-1* (*PBRM1*) gene, which are associated with chromatin remodelling are the most common alterations in ccRCC. Additionally, widespread DNA hypomethylation and metabolic shifts were identified. Based on comprehensive molecular studies, oncogenic metabolic shifts and epigenetic alterations have been considered the major pathogenic components of ccRCC.

The association of adipogenesis with ccRCC pathogenesis has been studied [9, 10]. ccRCC has a typical golden yellow cut surface on gross examination and consists of tumour cells with a clear to eosinophilic cytoplasm on histology [4]. These findings are due to the cellular accumulation of lipids and glycogen. Previous studies emphasized adipose differentiation-related protein (ADFP) and revealed that the adipogenesis pathway or adipogenic differentiation is associated with tumourigenesis and the prognosis of ccRCC [9, 10].

Plant homeodomain finger 2 (PHF2), in concert with CCATT/enhancer binding protein α (C/EBPα), plays a role in epigenetic regulation of adipogenesis [11-13]. These molecules induce transcription of genes related to adipogenesis by demethylating dimethylated histone H3 lysine 9 (H3K9me2). Alterations in PHF2 have been identified in several cancer types, including breast, oesophageal, stomach and colon cancer [14-16]. Additionally, C/EBPα has a role in tumourigenesis in various cancers, including acute myeloid leukaemia (AML) and cancers of the lungs, liver, breast, skin and ovaries [17-19]. Furthermore, both PHF2 and C/EBPα interact with p53 and act as tumour suppressors [16, 20]. However, the adipogenic role and association with the prognosis of ccRCC for PHF2 and C/EBPα have not yet been studied.

In this study, we postulated that the adipogenesis pathway with PHF2 and C/EBPα is associated with pathogenesis and prognosis of ccRCC. We evaluated adipogenesis by assessing the cytoplasmic features of ccRCC samples. Additionally, we assessed the nuclear expression of PHF2 and C/EBPα in ccRCC samples using immunohistochemistry. Additionally, we assessed the clinicopathological correlation with PHF2 and C/EBPα expression and evaluated the prognostic value. Furthermore, we proposed putative progression model of ccRCC based on our results.

## RESULTS

### Clinical and pathological characteristics of ccRCC patients

A total of 344 patients who underwent surgical resection and had confirmed ccRCC were analysed in this study. The patients included 251 men and 93 women. The mean age was 57 years old (range 20-80), and the average tumour size was 4.6 cm (range 0.5-20.0). Lymph node metastasis was found in 12 cases (3.5%), and distant metastasis was identified in 37 cases (10.8%). Of the 344 patients, 253 were categorized as stage I (73.5%), 28 stage II (8.1%), 24 stage III (7.0%) and 39 stage IV (11.3%). The WHO/ISUP grading scale revealed that 2 of the cases were grade 1 (0.6%), 169 cases were grade 2 (49.1%), 142 cases were grade 3 (41.3%) and 31 cases were grade 4 (9.0%). During follow-up, disease progression was found in 60 cases (17.4%) and cancer-related death was identified in 43 cases (12.5%). The mean follow-up period was 71.8 months (from 1 to 127 months, median 76.5 months).

### Nuclear expression level of PHF2 and C/EBPα in ccRCC

**Figure 1:**
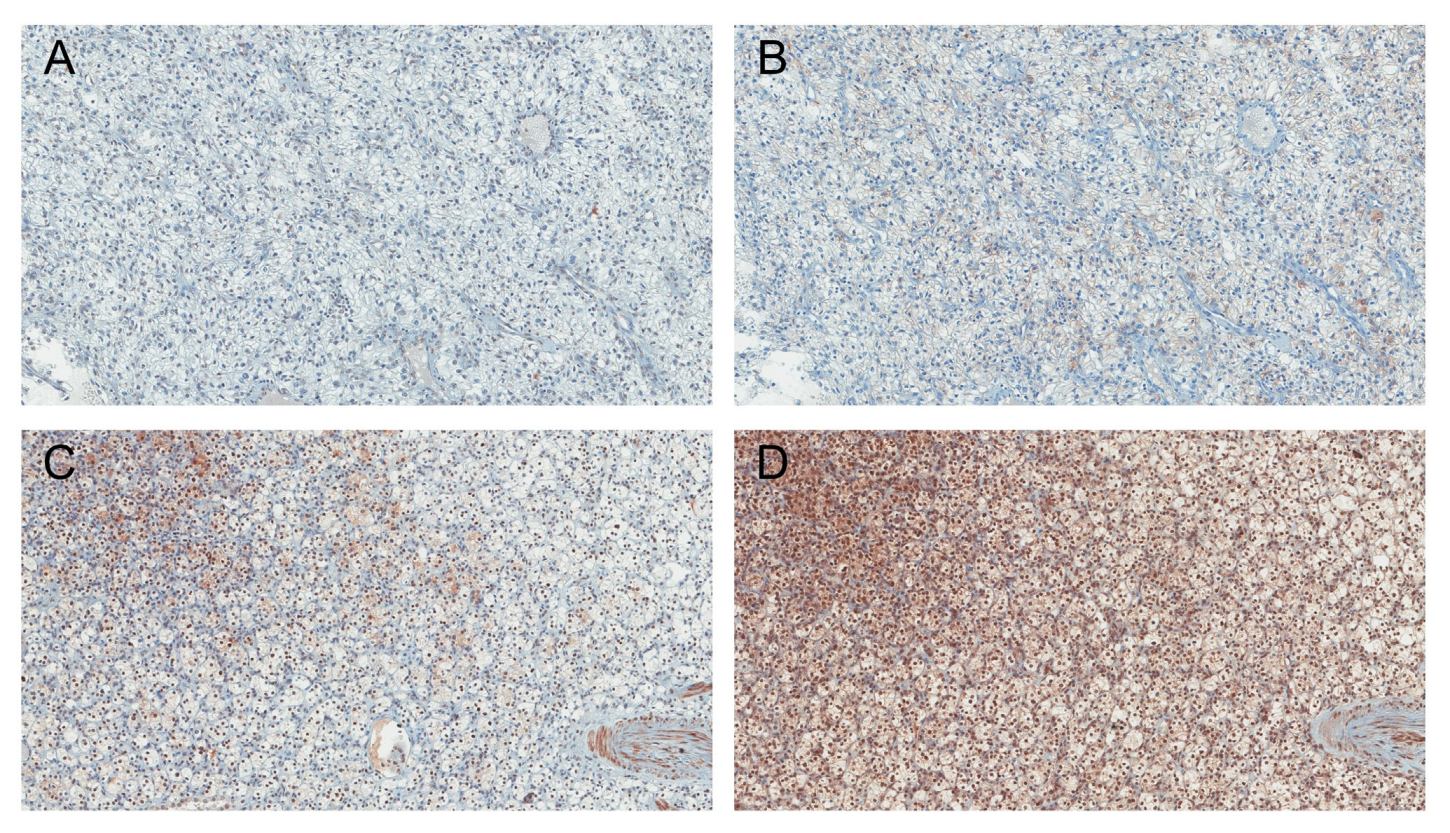
Immunohistochemical findings showing low A., B. and high C., D. nuclear expression of PHF2 and C/EBPα in ccRCC (A., C. PHF2 and B., D. C/EBPα) Original magnification, ×100.

The nuclear expression level of PHF2 and C/EBPα in tumour cells was evaluated (Figure 1), and 187 (54.4%) were classified as low expression and 157 (45.6%) were high expression. The PHF2 and C/EBPα expression were correlated (*P* < 0.001, Supplementary Table 1). Also, correlation between each protein and WHO/ISUP grade, pTNM stage and cytoplasmic feature was identified (Supplementary Table 2). In non-neoplastic kidney tissues, we identified moderate to strong immunoreactivity of PHF2 as cytoplasmic or nuclear staining and moderate immunoreactivity of C/EBPα as cytoplasmic staining on tubular epithelial cells.

### Cytoplasmic features of ccRCC and correlation with PHF2 and C/EBPα expression

The cytoplasmic features of ccRCC based on adipogenesis revealed that 200 cases had a clear cytoplasm (58.1%), 119 cases had an eosinophilic cytoplasm (34.6%) and 25 cases had high grade features (7.3%). Low expression of PHF2 and C/EBPα was significantly associated with higher cytoplasmic features (Table 1).

**Table 1:** Clinicopathologic features of patients with ccRCC and correlation between nuclear PHF2 and C/EBPα expression and clinicopathologic parameters.

	Nuclear expression of PHF2 and C/EBPα		
	Low (n = 187)	High (n = 157)	
	N (%)	N (%)	*P* value
Age (years)			**0.083**
≤ 57 yrs	82 (43.9)	84 (53.5)	
> 57 yrs	105 (56.1)	73 (46.5)	
Gender			**0.396**
Female	47 (25.1)	46 (29.3)	
Male	140 (74.9)	111 (70.7)	
Tumour size (cm)			**< 0.001**
≤ 7 cm	139 (74.3)	140 (89.2)	
> 7 cm	48 (25.7)	17 (10.8)	
WHO/ISUP grade			**0.003**
Grade 1 / 2	79 (42.2)	92 (58.6)	
Grade 3 / 4	108 (57.8)	65 (41.4)	
T stage			**0.003**
T 1	131 (70.1)	132 (84.1)	
T 2	32 (17.1)	9 (5.7)	
T 3	18 (9.6)	15 (9.6)	
T 4	6 (3.2)	1 (0.6)	
N stage			**0.073**
N0/Nx	177 (94.7)	155 (98.7)	
N1	10 (5.3)	2 (1.3)	
M stage			**0.003**
M0	158 (84.5)	149 (94.9)	
M1	29 (15.5)	8 (5.1)	
Stage			**0.003**
I	124 (66.3)	129 (82.2)	
II	20 (10.7)	8 (5.1)	
III	13 (7.0)	11 (7.0)	
IV	30 (16.0)	9 (5.7)	
Cytoplasm			**< 0.001**
clear	92 (49.2)	108 (68.8)	
eosinophilic	73 (39.0)	47 (29.9)	
high grade	22 (11.8)	2 (1.3)	

### Correlation of PHF2 and C/EBPα expression with clinicopathological parameters

The correlations of the nuclear expression of PHF2 and C/EBPα with clinicopathological parameters are shown in Table 1. Low expression was significantly associated with a larger tumour size, higher WHO/ISUP grade, high pM stage and advanced pTNM stage. Additionally, ccRCC with low expression had a marginally significant correlation with lymph node metastasis.

### Association of PHF2 and C/EBPα expression with prognosis

**Figure 2:**
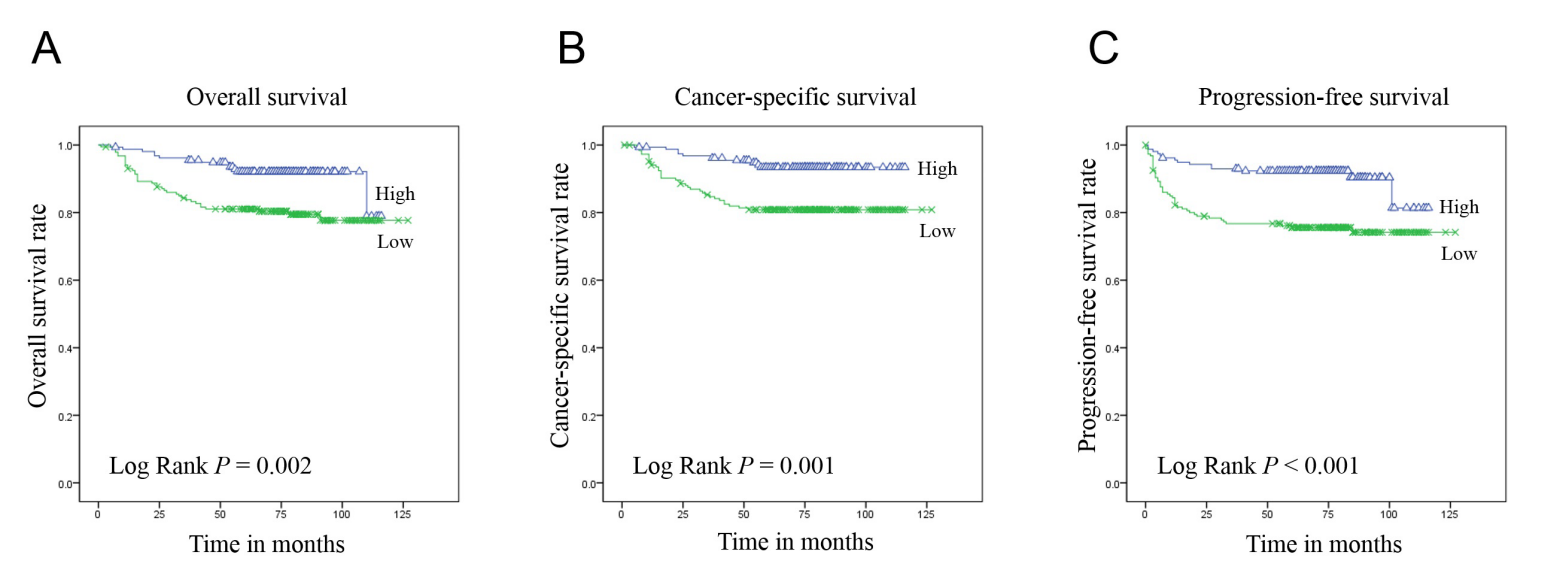
Kaplan-Meier curves of overall A., cancer-specific B. and progression-free C. survival in 344 patients with ccRCC according to the 2-tiered classification of PHF2 and C/EBPα nuclear expression

The PHF2 and C/EBPα expression levels had a significant correlation with the overall, cancer-specific and progression-free survival (Figure 2). The low expression group had significantly shorter overall, cancer-specific and progression-free survival periods than the high expression group. Furthermore, multivariate analysis using the Cox proportional hazards model indicated that the PHF2 and C/EBPα expression levels were an independent predictor of cancer-specific and progression-free survival in patients with ccRCC when assessed by the WHO/ISUP grade and pTNM stage (Table 2).

**Table 2:** Multivariate analysis of cancer-specific and progression-free survival with PHF2 and C/EBPα nuclear expression in 344 patients with ccRCC (Cox proportional hazard model).

Prognostic factors	Cancer-specific survival		Progression-free survival	
	Hazard ratio (95% CI)	*P* Value	Hazard ratio (95% CI)	*P* Value
WHO/ISUP grade		**0.002**		**< 0.001**
3, 4 versus 1, 2	6.752 (2.059 – 22.135)		4.332 (2.011 – 9.330)	
pTNM stage		**< 0.001**		**< 0.001**
III, IV versus I, II	21.500 (9.844 – 46.956)		12.223 (6.918 – 21.597)	
PHF2 and C/EBPα nuclear expression		**0.011**		**0.002**
Low versus High	2.491 (1.228 – 5.052)		2.596 (1.419 – 4.747)	

CI, Confidence interval.

## DISCUSSION

The association of adipogenesis with the ccRCC pathogenesis, clinicopathologic correlation and prognosis was investigated [9, 10]. One study examined the gene expression profiles of RCCs and demonstrated that *ADFP* gene was up-regulated in ccRCC compared to other RCC types and the normal kidney [9]. Additionally, real-time quantitative PCR revealed increased *ADFP* mRNA levels and immunohistochemistry for ADFP showed strong positivity in ccRCC. Moreover, a study revealed that strong *ADFP* immunohistochemical staining was associated with a low grade and low stage in ccRCC. The *ADFP* mRNA expression was higher in low grade than in high grade ccRCC. Univariate analysis showed that high *ADFP* expression was related to a better prognosis for cancer-specific survival, and high *ADFP* expression was identified as an independent prognostic factor of a better outcome by multivariate analysis. Another study revealed that ccRCC showed an adipogenic gene expression signature with *ADFP* gene up-regulation [10]. Additionally, an immunohistochemical study revealed increased ADFP expression and a differentiation experiment on ccRCC cell lines showed adipogenic transdifferentiation in adipogenic media. A study on early-stage ccRCC showed strong ADFP staining, which is consistent with a previous study that strong staining was associated the low stage [9]. These findings and a previous study [21] encouraged us to investigate the pathogenic role and clinicopathologic correlation between adipogenesis and ccRCC.

The epigenetic regulation of adipogenesis *via* histone modification has been elucidated [11-13]. Histones can be modified through various reactions, including ubiquitination, glycosylation, acetylation, phosphorylation and methylation [12]. Among various modifications, acetylation and methylation play a pivotal role in adipogenic differentiation. Additionally, the specific site of lysine (K) methylation affects the gene expression. For instance, methylation at H3K9 is associated with transcriptional silencing, whereas methylation at H3K4 is related to transcriptional activation. Recent studies have reported that PHF2, a JmjC family histone demethylase, has a role in metabolism, including adipogenesis [11-13]. Studies with *Phf2* knockout mice and PHF2 knock-down murine cell lines revealed that PHF2 is necessary for adipogenesis and PHF2 interacts with C/EBPα to demethylate H3K9me2 [11, 13]. These results suggested that PHF2, a histone demethylase, interacts with C/EBPα and regulates the expression of genes associated with adipogenesis by demethylating H3K9me2.

Furthermore, both PHF2 and C/EBPα have been identified to have tumour suppressive roles and interactions with p53 [14, 16-18, 22-24]. In breast cancer, alterations in the *PHF2* gene were observed in up to 60% of patients and reduction in the *PHF2* mRNA expression was identified [14]. Additionally, *PHF2* deletion or methylation showed a poor prognosis in the patient group > 40 years of age. For colon cancer, PHF2 expression was decreased in cancer tissue and the Oncomine database revealed PHF2 down-regulation in colon and stomach cancers [16]. Moreover, a colon and stomach cancer tissue study showed that PHF2 is positively correlated with p21 expression. In a study on HCT116 cancer cells, the authors showed that PHF2 is essential for p53 signalling pathway activation and demethylates H3K9me2 at p53 target promoters. Based on these findings, the authors suggested that PHF2 regulates p53-target gene expression by demethylating methylated H3K9 and showed that PHF2 acts as a tumour suppressor. The association between C/EBPα and cancer has been well documented in AML [17, 18]. C/EBPα is important for myeloid differentiation, and dysregulation of its expression is observed in AML and other haemato-lymphoid malignancies. In the lung, C/EBPα mRNA expression is down-regulated in lung cancer cell lines and low expression of C/EBPα has been identified in lung cancer patients by immunohistochemistry [22]. In a breast cancer study, low expression levels of C/EBPα mRNA and protein were identified, and induction of C/EBPα inhibited growth [23]. In murine squamous cell carcinoma of the skin, the C/EBPα mRNA and protein expression levels were decreased in cancer cell lines. Additionally, re-expression of C/EBPα inhibited cancer cell proliferation [24]. C/EBPα is thought to act as a tumour suppressor, which interacts with various molecules, including p21, CDK2, CDK4, E2F, SWI/SNF chromatin-remodelling complex, and p53 [18, 20, 25, 26]. These study results encouraged us to evaluate the adipogenic role and association with ccRCC progression of PHF2 and C/EBPα.

In this study, we assessed adipogenesis by evaluating the cytoplasmic features of ccRCC. For a precise study based on the PHF2 and C/EBPα function, evaluation of the methylation H3K9 status would be reasonable. We tried to assess the methylation status of H3K9 by immunohistochemistry. However, the H3K9me2 immunohistochemical study has some shortcomings in assessing adipogenesis. First, H3K9me2 immunohistochemical positivity may represent the presence of H3K9me2, but negativity may not represent demethylation of H3K9me2 and negative staining may be found in H3K9me1 or H3K9me3. Second, H3K9me2 immunostaining shows the global genomic H3K9 methylation status without representing the H3K9 methylation status of specific PHF2 and C/EBPα target regions. For these reasons, we assessed the cytoplasm to evaluate adipogenesis. As mentioned in the materials and methods section, cytoplasmic features were categorized as clear to light granular, deeply granular or eosinophilic, and high grade features (rhabdoid, sarcomatoid, and unclassifiable high grade morphology). Cases with different features were categorized as higher cytoplasmic features. Those cytoplasmic features were correlated with the nuclear expression of PHF2 and C/EBPα in consideration of the role of these proteins in adipogenesis (Table 1). Those results were consistent with previous reports that an eosinophilic cytoplasm is associated with high-grade tumours, necrosis or haemorrhage [4].

Molecular characteristics of ccRCC have been identified [7]. In TCGA data for ccRCC, genetic alterations in PHF2 and C/EBPα were found in a single patient case (0.2%) for each [7, 27, 28]. The patients had a *PHF2* missense mutation and *CEBPA* truncating mutation, respectively. Copy number alterations were not identified in either gene. The mRNA expression of *PHF2* was up-regulated in 11 patients (2.3%) and down-regulated in 28 patients (6.0%), and the *CEBPA* mRNA expression was up-regulated in 22 patients (4.7%), while down-regulation was not identified. Alterations in the protein expression were not identified. Those results showed that both the *PHF2* and *CEBPA* genes are mutated in a very small proportion of ccRCC patients and the mRNA expression of both genes was not constant. The TCGA datasets are not well correlated with our data; we observed low nuclear expression of PHF2 and C/EBPα in 187 patients (54.4%). These discrepancies can be explained by considering that epigenetic regulation affects PHF2 and C/EBPα expression for a low frequency of both gene mutations and mRNA expression is not well correlated with the protein expression or nuclear location of proteins or the protein function for different mRNA levels. Those possibilities should be assessed in integrative genomic levels in a comprehensive manner that considers transcriptional and translational modifications.

**Figure 3:**
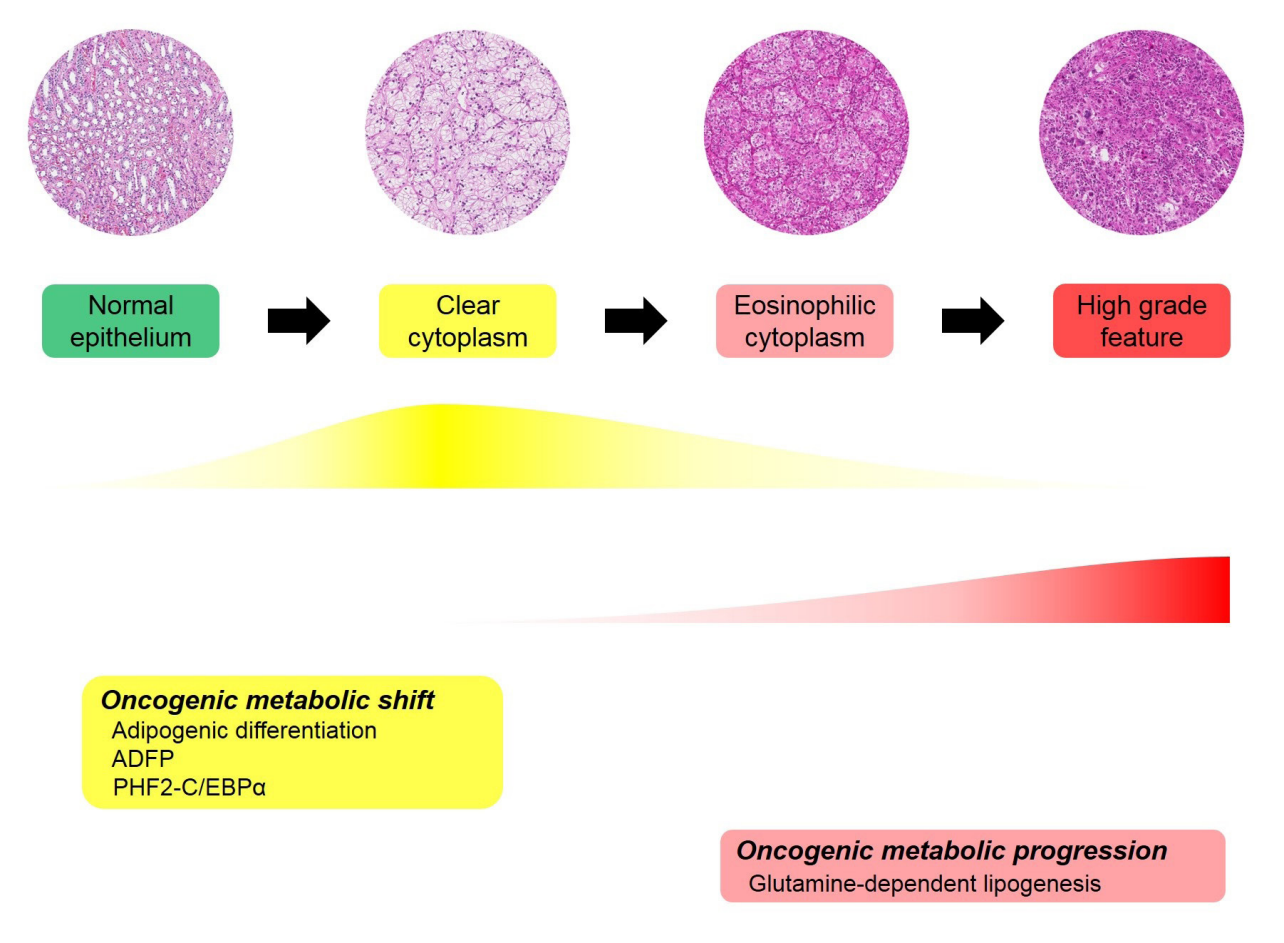
Proposed adipogenic metabolic evolution in ccRCC progression

Cancer cell metabolism has been at the forefront of cancer research, and metabolic changes in cancer cell have been widely studied [29-31]. Cancer cells require energy production and biosynthesis of macromolecules, including nucleotides, lipids and proteins. As *VHL* mutation is the most common genetic alteration in ccRCC and VHL affects cellular energy metabolism through HIF-1α, ccRCC cases have received substantial attention at the metabolic level [7, 32, 33]. A cancer metabolic study by NGS for DNA sequencing or transcriptome analysis revealed that a worse prognosis in ccRCC was correlated with up-regulation of fatty acid synthesis genes [7]. This includes increased acetyl-CoA carboxylase (ACC) protein and *fatty acid synthase* (*FASN*) mRNA levels. Metabolites analysis in ccRCC showed increased metabolite levels in fatty acid biosynthesis during tumourigenesis and reversal of this pattern during progression [33]. Additionally, the study revealed that medium chain fatty acids are decreased in late-stage tumours, which is consistent with the observation that the lipid content is decreased in high-grade ccRCC [34]. The results of mRNA sequencing and metabolite analysis were not well correlated in our study or in other studies. The previous studies assessed ADFP and our results suggested that adipogenesis is associated with tumour initiation and decreased adipogenic function of ADFP and PHF2 and C/EBPα with tumour progression [9, 10]. The discrepancy between decreased ADFP, PHF2 and C/EBPα expression and increased fatty acid synthesis genes in advanced ccRCC can be explained in several ways. First, ADFP, PHF2 and C/EBPα are regulatory proteins that are associated with adipogenesis, and they do not individually correlate with enzymes in fatty acid synthesis. Second, the enzyme levels, ACC and FASN levels can be affected by various molecules and conditions. Third, increased fatty acid synthesis genes do not indicate there is increased adipogenesis and may be a result of the negative feedback of products. Indeed, as mentioned before, medium chain fatty acids are decreased in advanced ccRCC in metabolite analysis [33]. These raised the possibility of another adipogenic pathway in ccRCC metabolism. Additionally, transcriptome analysis showed that poor survival is correlated with decreased Krebs cycle activity (tricarboxylic acid cycle, TCA cycle) [7]. Metabolite analysis revealed that glucose, citrate and glutamate are increased in ccRCC cancer cells compared to normal tissue and there is a decreased citrate level in high stage ccRCC compared to low stage ccRCC [33]. Based on another possible adipogenic pathway, increased glutamate in cancer and a decreased citrate level in high-stage ccRCC led us to consider adipogenic metabolic evolution. We suggested sequential evolution based on adipogenesis in ccRCC (Figure 3). In our hypothetical adipogenic metabolic evolution, ccRCC cancer cells undergoing tumourigenesis are in a sufficient energy state due to the VHL/HIF pathway. Additionally, fertile energy leads to physiologic adipogenesis with ADFP, PHF2 and C/EBPα overexpression. This status can be called an oncogenic metabolic shift. As tumour progresses, glutamine-dependent lipogenesis is dominant and previous physiologic adipogenesis is decreased. Advanced cancer does not reach the energy requirement, which would further affect metabolic alterations. Additionally, the precise regulation of metabolism would be disrupted due to the accumulation of genetic alterations. Additionally, because PHF2 and C/EBPα are considered to have a tumour suppressive effect, subclones of cancer that suppress PHF2 and C/EBPα would have a survival advantage in tumour progression. As adipogenic metabolism is switched from the physiologic pathway to a Warburg-like effect, cancer cells become more pleomorphic and aggressive and have a non-clear morphology. Additionally, we named this status ‘oncogenic metabolic progression’ when considering the specific condition of ccRCC and difference from the oncogenic metabolic shift. Our hypothesis can explain our results and previous studies; however, more detailed evaluation and studies are needed for confirmation.

In summary, we evaluated the clinicopathologic correlation and prognostic significance of nuclear PHF2 and C/EBPα expression in ccRCC using an immunohistochemical study. We demonstrated that a low expression level of PHF2 and C/EBPα was significantly correlated with a larger tumour size, higher WHO/ISUP grade, high pM stage, advanced pTNM stage, and shorter overall, cancer-specific and progression-free survival times. Multivariate analysis showed that PHF2 expression and C/EBPα expression are independent prognostic factors for cancer-specific and progression-free survival and could act as novel prognostic markers in ccRCC patients. Furthermore, cytoplasmic features were associated with the PHF2 and C/EBPα expression and patient prognosis. Based on the adipogenic role of PHF2 and C/EBPα, we suggested adipogenic metabolic evolution and our consideration may elucidate a therapeutic strategy for cancer cell metabolism.

## MATERIALS AND METHODS

### Patients and clinicopathologic information

A total of 344 patients (251 men, 93 women) with ccRCC who underwent radical or partial nephrectomy between January 1, 2005, and March 31, 2008, at Seoul National University Hospital were included in this retrospective study. Each ccRCC sample was evaluated according to the RCC histologic type, WHO/ISUP grade and tumour stage. WHO/ISUP grades were reviewed based on the description in Delahunt *et al.* [35]. Tumour staging was re-assessed according to the 2010 TNM classification system [6] by reviewing the gross description, gross photos and representative slides. Tumour recurrence or distant metastasis of ccRCC was determined according to the clinical and radiologic findings. Disease-related deaths were verified by reviewing the patients’ medical records. This study was approved by the Institutional Review Board of Seoul National University Hospital (IRB No. 1609-020-789).

### Tissue microarray (TMA)

The haematoxylin and eosin (H & E stain) slides from 344 ccRCC patients were reviewed. For TMA construction, a sufficient viable tumour portion with no haemorrhage or necrosis was selected in each case. Two representative core sections (2 mm in diameter), one for dominant histologic features and the other for high grade areas, were taken from formalin-fixed paraffin blocks because of the tumour heterogeneity. They were embedded in new recipient paraffin blocks (TMA blocks) using a trephine apparatus (Superbiochips Laboratories, Seoul, Korea). Additionally, cortical and medullary portions of 15 non-neoplastic kidney tissues from ccRCC patients were included as a control group.

### Evaluation of the cytoplasmic features of ccRCC

**Figure 4:**
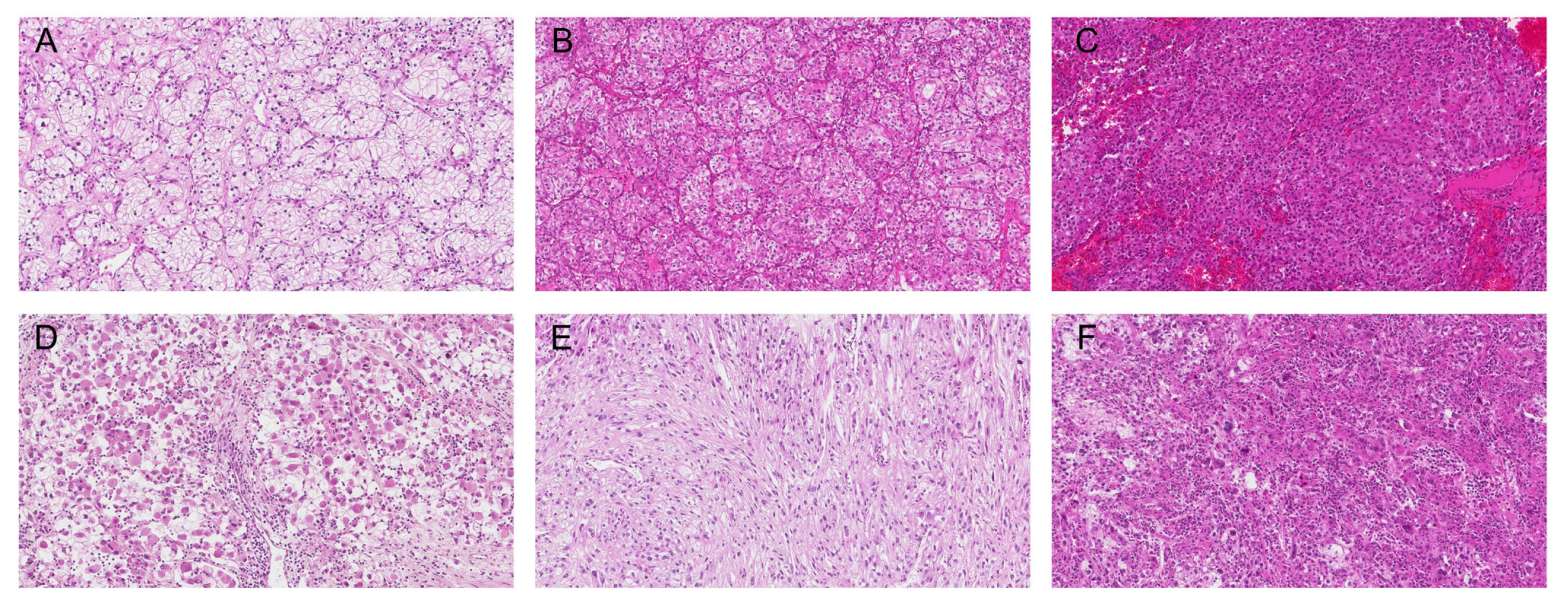
Cytoplasmic features of ccRCC based on adipogenesis **A.** Clear to light granular, **B.**-**C.** deeply granular or eosinophilic, and **D.**-**F.** high grade features (rhabdoid **D.**, sarcomatoid **E.**, and unclassifiable high grade morphology **F.**). Original magnification, ×100.

H & E staining was performed on 4 μm-thick sections taken from the TMA blocks. Cytoplasmic features based on adipogenesis were evaluated in 3 categories. We first categorized as clear cytoplasm *versus* non-clear cytoplasm. Further, we classified non-clear group into non-clear cytoplasm with low WHO/ISUP grade *versus* non-clear cytoplasm with high grade features (rhabdoid, sarcomatoid, and unclassifiable high grade morphology). Finally, we grouped into clear to light granular, deeply granular or eosinophilic, and high grade features (Figure 4). Each core was evaluated and cases with different cytoplasmic features were categorized into a higher grade category (Supplementary Figure 1).

### Immunohistochemistry of PHF2 and C/EBPα

The immunohistochemical staining of PHF2 and C/EBPα was conducted on 4-μm thick sections collected from the TMA slides. TMA slides were treated to remove wax and rehydrated in a graded series of alcohol solutions. Immunohistochemical staining was performed using the Ventana BenchMark XT (Ventana Medical Systems, Tucson, AZ). Each polyclonal rabbit anti-PHF2 antibody (Novus Biologicals, Littleton, CO) and polyclonal rabbit anti-C/EBPα antibody (Santa Cruz Biotechnology, Dallas, TX) was diluted 1:200. After the heat-induced antigen retrieval, the primary antibody was incubated with the samples for 15 minutes. The binding of the primary antibody was identified using the OptiView DAB IHC Detection Kit (Ventana Medical Systems) according to the manufacturer’s instructions.

### Interpretation of TMA immunohistochemistry

The nuclear staining of PHF2 and C/EBPα was assessed based on epigenetic regulation of adipogenesis of both antibodies. Immunoreactivity was semi-quantitatively evaluated by assessing the intensity and extent of nuclear staining. The intensity of immunostaining was sorted into negative, weak, moderate, and strong. The extent of immunostaining was divided into 0%, less than or equal to 10%, greater than 10 to less than or equal to 50%, and greater than 50%. Collectively, immunoreactivity with moderate to strong intensity an extent of greater than 10% is classified as positive staining and immunoreactivity with weak staining with any extent and moderate to strong intensity an extent of less than 10% negative staining. The cutoff value 10% showed the most reliable results. For each antibody, cases that were negative in both cores were designated as having low expression and cases that were positive in at least one core were classified as having high expression. Finally, cases with low expression in PHF2 and/or C/EBPα were classified as the low expression group, whereas cases with high expression in both PHF2 and C/EBPα were classified as the high expression group (Supplementary Figure 2).

### Statistical analysis

The relationship between the nuclear expression of PHF2 and C/EBPα and adipogenesis was analysed with the Pearson’s χ^2^ test. Correlations between their expression level and clinicopathological parameters were analysed by the Pearson’s χ^2^ and Fisher exact tests. The progression-free survival period was determined as the interval between the primary radical or partial nephrectomy and the final follow-up visit or identification of recurrence or metastasis of the ccRCC. The cancer-specific survival period was defined by the interval between the primary radical or partial nephrectomy and the final follow-up visit or cancer-related death. For survival analyses, the Kaplan-Meier curve and log-rank test were applied, and the univariate analysis of overall, cancer-specific, or progression-free survival was performed. A Cox proportional hazard model was applied for the multivariate analysis. In all statistical analyses, a two-tailed *P* < 0.05 was considered statistically significant. All statistical analyses were performed using IBM SPSS Statistics 21 (IBM SPSS Inc., Chicago, IL, USA).

## SUPPLEMENTARY MATERIALS FIGURES AND TABLES



## Abbreviations

ADFP: adipose differentiation-related protein
AML: acute myeloid leukaemia
ccRCC: clear cell renal cell carcinoma
C/EBPα: CCATT/enhancer binding protein α
*FASN*: *fatty acid synthase*
HIF: hypoxia inducible factor
NGS: next-generation sequencing
*PBRM1*: *polybromo-1*
PHF2: Plant homeodomain finger 2
RCC: renal cell carcinoma
*VHL*: *Von Hippel-Lindau*
