# Supplementary material for: The prognostic significance of nuclear expression of PHF2 and C/EBPα in clear cell renal cell carcinoma with consideration of adipogenic metabolic evolution
Source: Oncotarget. 2017 Aug 4;9(1):142–51. doi: 10.18632/oncotarget.19949 (PMC5787448; doi:10.18632/oncotarget.19949)
Supplement: Supplementary file 1 [file oncotarget-09-142-s001.pdf]

The prognostic significance of nuclear expression of PHF2 and C/EBPα in clear cell renal cell carcinoma with consideration of adipogenic metabolic evolution

SUPPLEMENTARY MATERIALS

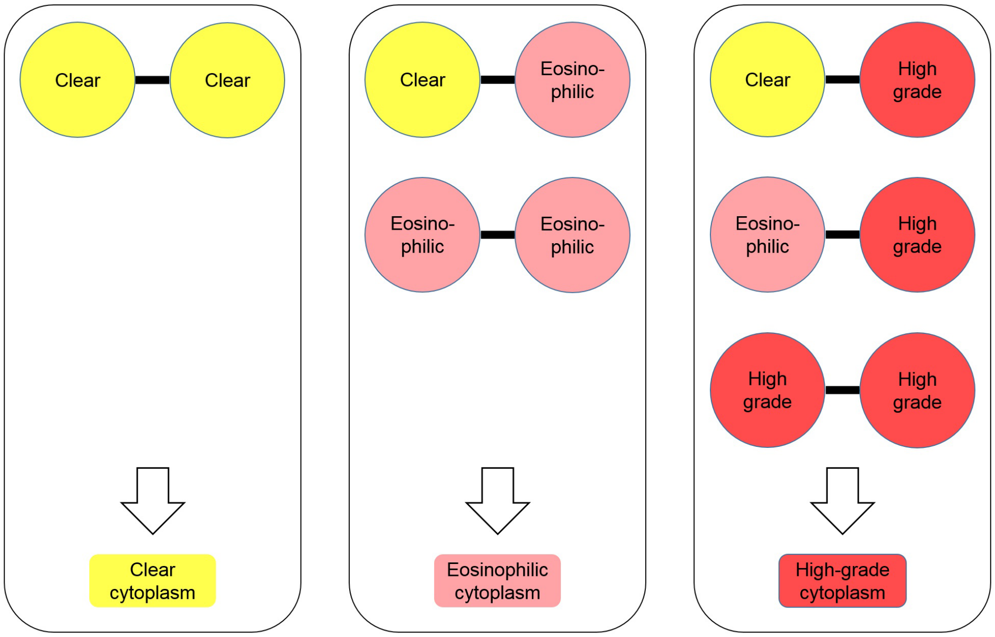

Supplementary Figure 1: Assessment and categorization of cytoplasmic features.

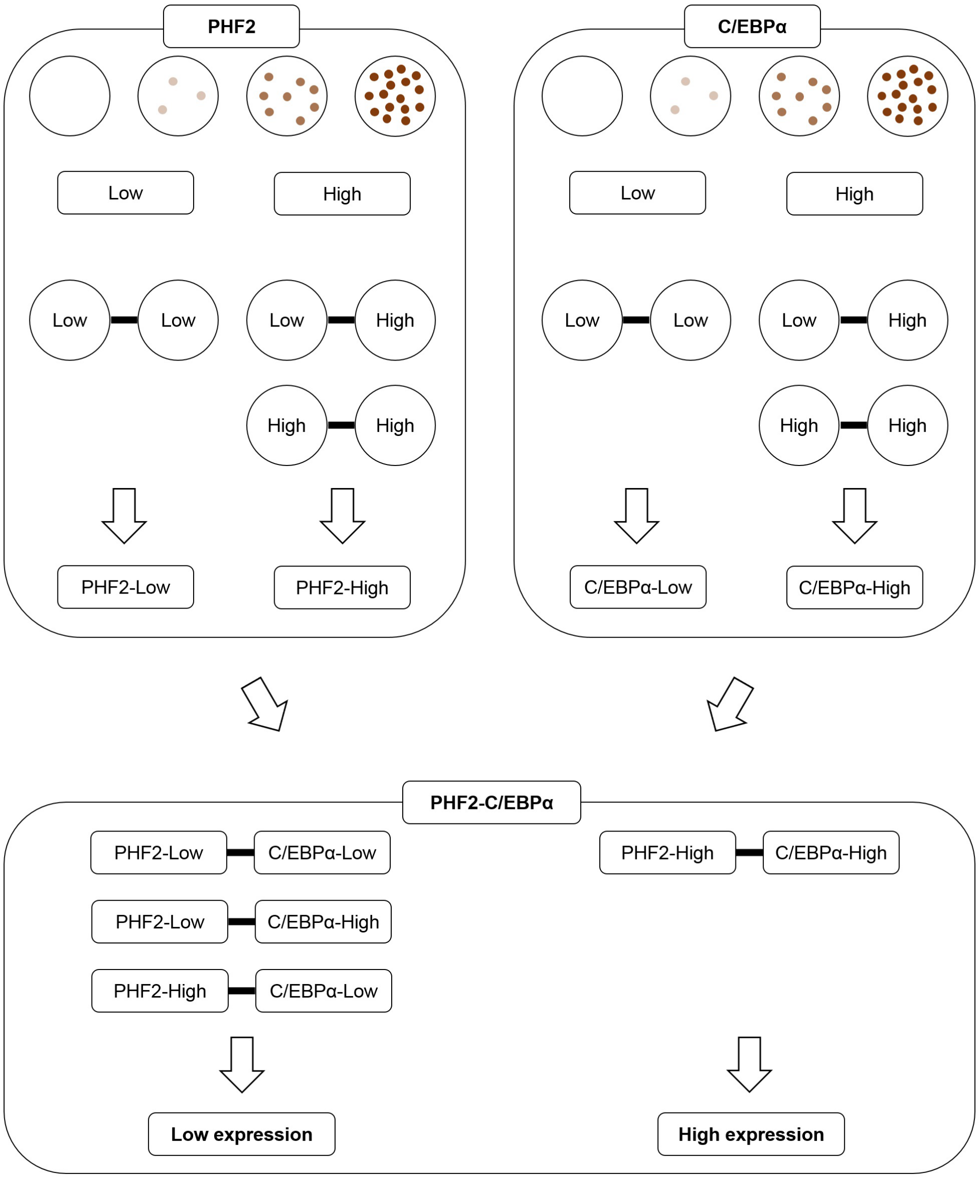

Supplementary Figure 2: Assessment of PHF2 and C/EBPα nuclear expression and categorization of PHF2-C/EBPα expression level.

Supplementary Table 1: Correlation between nuclear expression of PHF2 and C/EBPα

	PHF2		
	Low (n = 171)	High (n = 173)	
	N (%)	N (%)	*P* value
C/EBPα			**< 0.001**
Low (n= 59)	43 (25.1)	16 (9.2)	
High (n= 285)	128 (74.9)	157 (90.8)	

Supplementary Table 2: Correlation between nuclear PHF2 and C/EBPα expression and clinicopathologic parameters

	Nuclear expression of PHF2			Nuclear expression of C/EBPα		
	Low (n = 171)	High (n = 173)		Low (n = 59)	High (n = 285)	
	N (%)	N (%)	*P* value	N (%)	N (%)	*P* value
Age (years)			**0.334**			**0.021**
≤ 57 yrs	78 (45.6)	88 (50.9)		20 (33.9)	146 (51.2)	
> 57 yrs	93 (54.4)	85 (49.1)		39 (66.1)	139 (48.8)	
Gender			**0.333**			**0.872**
Female	42 (24.6)	51 (29.5)		15 (25.4)	78 (27.4)	
Male	129 (75.4)	122 (70.5)		44 (74.6)	207 (72.6)	
Tumour size (cm)			**0.001**			**0.003**
≤ 7 cm	126 (73.7)	153 (88.4)		39 (66.1)	240 (84.2)	
> 7 cm	45 (26.3)	20 (11.6)		20 (33.9)	45 (15.8)	
WHO/ISUP grade			**0.001**			**0.010**
Grade 1 / 2	70 (40.9)	101 (58.4)		20 (33.9)	151 (53.0)	
Grade 3 / 4	101 (59.1)	72 (41.6)		39 (66.1)	134 (47.0)	
T stage			**0.005**			**0.085**
T 1	119 (69.6)	144 (83.2)		38 (64.4)	225 (78.9)	
T 2	29 (17.0)	12 (6.9)		12 (20.3)	29 (10.2)	
T 3	17 (9.9)	16 (9.3)		7 (11.9)	26 (9.1)	
T 4	6 (3.5)	1 (0.6)		2 (3.4)	5 (1.8)	
N stage			**0.020**			**0.008**
N0/Nx	161 (94.2)	171 (98.8)		53 (89.8)	279 (97.9)	
N1	10 (5.8)	2 (1.2)		6 (10.2)	6 (2.1)	
M stage			**0.003**			**0.005**
M0	144 (84.2)	163 (94.2)		46 (78.0)	261 (91.6)	
M1	27 (15.8)	10 (5.8)		13 (22.0)	24 (8.4)	
Stage			**0.006**			**0.020**
I	113 (66.1)	140 (80.9)		34 (57.6)	219 (76.8)	
II	18 (10.5)	10 (5.8)		7 (11.9)	21 (7.4)	
III	12 (7.0)	12 (6.9)		6 (10.2)	18 (6.3)	
IV	28 (16.4)	11 (6.4)		12 (20.3)	27 (9.5)	
Cytoplasm			**< 0.001**			**< 0.001**
clear	83 (48.5)	117 (67.6)		22 (37.3)	178 (62.4)	
eosinophilic	68 (39.8)	52 (30.1)		24 (40.7)	96 (33.7)	
high grade	20 (11.7)	4 (2.3)		13 (22.0)	11 (3.9)	
